# Retrospective analysis of the impact of dose delay and reduction on outcomes of colorectal cancer patients treated with FOLFIRI‑based treatment

**DOI:** 10.7717/peerj.15995

**Published:** 2023-09-12

**Authors:** Xia Zhang, Hongjuan Zheng, Cheng Cai, Yinzi Xu, Mengzhen Xie, Qinghua Wang, Xiayun Jin, Jianfei Fu

**Affiliations:** 1 Department of Medical Oncology, Affiliated Jinhua Hospital, Zhejiang University School of Medicine, Jinhua, China; 2 Department of Colorectal Surgery, Affiliated Jinhua Hospital, Zhejiang University School of Medicine, Jinhua, China; 3 Department of Pathology, Second Affiliated Hospital of Zhejiang University School of Medicine, Hangzhou, China

**Keywords:** Dose delay, Dose reduction, Relative dose intensity (RDI), Metastatic colorectal cancer, FOLFIRI

## Abstract

**Objectives:**

To determine the relationship between chemotherapy dose delay/reduction with progression-free survival (PFS) and overall survival (OS) in colorectal cancer patients treated with FOLFIRI based first-line chemotherapy in real-world retrospectively study.

**Methods:**

We identified 144 eligible patients with advanced CRC who received FOLFIRI as first-line based treatment. The study protocol was submitted to the institutional review board and was exempted. Dose delay was defined as an average delay of more than 3 days (>3 days *vs*. ≤3 days) from the intended date. Dose reduction (actual dose/standard dose * 100%) ≤85% was considered as chemotherapy reduction in the chemotherapy dose relative to the standard (mg/m^2^) regimen for all cycles. Relative dose intensity (RDI) ≤80% was described as chemotherapy reduction. OS and PFS were measured using Kaplan–Meier and Cox proportional hazard models.

**Results:**

There were 114 patients with chemotherapy dose delay (dose delay >3 days). PFS of patients without dose delay had better survival than patients with dose delay (*p* = 0.002). There were 28.47% patients treated with dose reduction of 5-Fu. PFS and OS were better in patients without 5-Fu dose reduction than in patients with 5-Fu dose reduction with *p* values of 0.024 and <0.001, respectively. Patients with high 5-FU RDI had better PFS than patients with low 5-FU RDI (*p* < 0.001). While, there was no statistical difference in OS between the two groups. Then we stratified the analysis by age. In <65 years cohort, both PFS and OS were better in patients with high 5-Fu RDI than in those with low 5-Fu RDI (*p* < 0.001, *p* = 0.005, respectively). But, in ≥65 years cohort, OS were better in patients with low 5-Fu RDI than in those with high 5-Fu RDI (*p* = 0.025). Moreover, both dose reduction and RDI of irinotecan had no statistically significant difference in both PFS and OS.

**Conclusion:**

In the advanced colorectal cancer patients who received FOLFIRI based treatment as first-line regimen, chemotherapy dose delay and reduction dose of 5-Fu were associated with worse survival, especially among patients younger than 65 years.

## Background

Colorectal cancer (CRC) is one of the leading causes of cancer death worldwide. Survival among patients with metastatic colorectal cancer (mCRC) has been improved since the addition of monoclonal antibodies to vascular endothelial growth factor (VEGF) or endothelial growth factor receptor (EGFR). Irinotecan and oxaliplatin added to 5-fluorouracil/leucovorin therapy remain the key drugs in the treatment of patients with mCRC (Innocenti et al., 2021). At present, irinotecan‑based chemotherapies combined with cetuximab or bevacizumab are standard treatment regimens for mCRC (Elez, Argilés & Tabernero, 2015).

The importance of a high relative dose intensity (RDI) for survival has been established for some cancers, including breast cancer (Lyman, Dale & Crawford, 2003), ovarian cancer (Anuradha et al., 2016) and lymphoma (Lyman et al., 2004); however, information about the effects of a high RDI among patients with CRC is limited. A high RDI is associated with improved survival, especially for patients with breast cancer or non-Hodgkin’s lymphoma (NHL) (Lyman, Dale & Crawford, 2003; Lyman et al., 2004). An RDI below 70–85% is generally considered to be a clinically meaningful threshold for reduction in the RDI based on previously published studies (Hanna et al., 2013; Lyman, 2009; Aspinall et al., 2015; Crawford et al., 2020). In the adjuvant setting, the importance of receiving full-dose chemotherapy on time has been well established (Aspinall et al., 2015; Gray et al., 2019; Denduluri et al., 2015). However, clinical goals differ from adjuvant settings, which include control of cancer growth, symptom management and extension of life in advanced disease. Chemotherapy dose delay and reduction are common in advanced disease to manage haematologic and infectious side effects or surgery (Crawford et al., 2020; Denduluri et al., 2018). In addition, there are nonmedical indications for delays, such as holidays, poor compliance and personal schedule preferences (Kogan, Davis & Brooks, 2019). Such agents are commonly associated with toxicities, leading to the occurrence of dose delay and reduction, which contribute to reduced RDI.

Dose delay and reduction occur more frequently in real-world populations than in more selected clinical trial populations, in which the benefits of these regimens were initially tested. However, the impact of chemotherapy modification on survival has not been well confirmed in the real-world setting. In addition, there are no established recommendations for delay time or dose reduction. Therefore, the purpose of this analysis was to retrospectively analyse the effect of dose delay/reduction on progression-free survival (PFS) and overall survival (OS) among patients with mCRC receiving FOLFIRI as first-line chemotherapy.

## Methods

### Study setting and population

The inclusion criteria were as follows: (1) patients diagnosed with advanced colorectal cancer; (2) patients at The Affiliated Jinhua Hospital, Zhejiang University School of Medicine from July 1, 2017, to February 1, 2022, or at the Second Affiliated Hospital of Zhejiang University School of Medicine from December 1, 2018, to December 31, 2020; (3) patients received 4 to 12 cycles of FOLFIRI-based treatment as a first-line regimen; (4) the age at diagnosis was limited from 28 to 83.

The exclusion criteria were as follows: (1) patients receiving fewer than four cycles of FOLFIRI-based treatment, as these patients may have had an inconsistent effect on treatment outcomes (PFS and OS) due to relative chemoresistance or personal reasons; (2) patients who accepted XELIRI, FOLFOX, XELOX or FOLFIRINOX as first-line regimens; (3) patients who progressed within 6 months after oxaliplatin‑based adjuvant chemotherapy; FOLFIRI-based treatment was considered a second-line treatment; (4) patients who received part of the FOLFIRI cycles in another hospital, because we cannot confirm the exact time and dose of each chemotherapy cycle for these patients; and (5) patients with multiple primary tumours.

### Data sources and data collection

Data were extracted from our electronic medical record (EMR) and our Guide Patients Support care (GPS) database. The GPS database is a web-based electronic treatment follow-up record system with oncology-specific data. We received a waiver for the need for informed consent from participants in our study. We identified 135 eligible patients with advanced CRC who received FOLFIRI as a first-line treatment (July 1, 2017–February 1, 2022) at the Affiliated Jinhua Hospital, Zhejiang University School of Medicine. We also identified nine eligible patients with advanced CRC who received FOLFIRI as a first-line treatment (December 1, 2018–December 31, 2020) at the Second Affiliated Hospital of Zhejiang University School of Medicine. And this study was approved by the ethics committees of Affiliated Jinhua Hospital, Zhejiang University School of Medicine (Approval Number: 2022LSD89, Zhejiang, China) and Second Affiliated Hospital of Zhejiang University School of Medicine (Approval Number: 2023LSYD0691, Zhejiang, China).

We retrieved records of year and age at diagnosis, time of chemotherapy, dose of chemotherapy, sex, height and weight, Nutrition Risk Screening 2002 (NRS2002), ECOG score, comorbidity, tumour location, histological type, differentiated grade, T-classification, N-classification, stage TNM, operation, liver metastasis, number of metastatic organs, administration of radiotherapy, administration of chemotherapy and target therapy (including bevacizumab and cetuximab), RAS/BRAF mutation status, HER2 and MSI status, continuity of chemotherapy, treatment effect (overall response rate, ORR), platelet count, white blood cell count, neutrophil count, haemoglobin, glutamic-pyruvic transaminase, glutamic-oxalacetic transaminase, total bilirubin, time of progression and time of death.

In addition, the doses and dates of administration were recorded for every cycle. The unit of analysis was the first-line chemotherapy course for each patient. The actual delivered number of chemotherapy cycles, actual delivered dose in each cycle, and actual length of each cycle were recorded.

#### Variable declaration

According to the study of Neugut et al. (2006), age could be regrouped as <65 years old and ≥65 years old. According to the NRS2002 score, patients were divided into the ‘without undernutrition’ group (NRS2002 < 3) and the ‘with undernutrition’ group (NRS2002 ≥ 3). Tumour location was divided into left colon cancer and right colon cancer. Histological type was grouped as adenocarcinoma, mucous adenocarcinoma and unknown. The differentiated grade was stratified into moderate, poor and unknown. The operation and liver metastasis were grouped as NO and YES. Number of metastatic organs divided into <2 and ≥2. The RAS and BRAF genes were stratified into wild-type, mutation and unknown. The HER2 gene was stratified into negative, positive and unknown. Microsatellite instability (MSI) was stratified into microsatellite stability (MSS), MSI-high and unknown. Bevacizumab and cetuximab were classified as NO and YES according to the combination of targeted therapies. The overall response rate (ORR) was classified as NO, YES and unknown. The radiotherapy variable was classified as NO and YES. Platelets (PLTs), white blood cells (WBCs), neutrophils (NEUs), haemoglobin (Hb), glutamic-pyruvic transaminase (ALT), glutamic-oxalacetic transaminase (AST) and total bilirubin (TBil) were grouped as normal and abnormal.

### Dose delay, dose reduction and relative dose intensity

Chemotherapy completion was defined as all standard doses of chemotherapy. Dose delay was defined as an average delay of more than 3 days (>3 days *vs*. ≤3 days) (Kogan, Davis & Brooks, 2019; Cespedes Feliciano et al., 2017; Fauci et al., 2011) from the intended date. The total dose received and dose intensities for irinotecan and 5-Fu were calculated for each patient. Dose reduction (actual dose/standard dose * 100%) ≤85% was considered chemotherapy reduction. RDI [(actual dose * 14)/(standard dose * (dose delay + 14)) * 100%] ≤80% was also described as chemotherapy reduction (low RDI).

### Chemotherapy regimens

FOLFIRI consisted of leucovorin 400 mg/m^2^/d and irinotecan 180 mg/m^2^ as a 2-h infusion on day 1 followed by bolus 5-Fu, 400 mg/m^2^ then by a 46-h 5-Fu infusion of 2,400 mg/m^2^. This cycle was repeated every 2 weeks for 4–12 cycles.

### Statistical analysis

OS and PFS were plotted using the Kaplan‒Meier method. The distribution of clinicopathological characteristics between the low 5-Fu RDI group and the high 5-Fu RDI group was analysed using chi-squared tests. The primary endpoint was progression-free survival (PFS), which was calculated from the date of first-line FOLFIRI chemotherapy to the date of progression. The secondary endpoint was overall survival (OS), which was calculated from the date of FOLFIRI-based first-line treatment to the date of death. Survival curves were generated using the Kaplan‒Meier method, and the log-rank test was carried out to evaluate the survival differences between groups.

We used R 4.0.3 software (R Core Team, 2020) for statistical analysis. When the bilateral *p* value < 0.05, the difference was considered statistically significant.

## Results

### Clinicopathological characteristics of patients with advanced CRC treated with FOLFIRI-based first-line chemotherapy

We identified 144 eligible patients with advanced CRC who received FOLFIRI-based first-line treatment. There were 124 (86.11%) patients treated with reduced doses of 5-Fu (5-Fu RDI ≤ 80%, low Fu-RDI group) and 20 (13.89%) patients treated with nonreduced doses of 5-Fu (high Fu-RDI group). There were basically no significant differences in clinicopathological characteristics between the two groups, and detailed information is shown in Table 1.

**Table 1:** The characteristics of patients with advanced colorectal cancer treated with FOLFIRI based first-line treatment.

Characteristic	Number	Low Fu-RDI (%)	High Fu-RDI (%)	*P* ^a^
**Total**	144	124 (86.11)	20 (13.89)	
**Gender**				0.733
Male	96	82 (66.13)	14 (70)	
Female	48	42 (33.87)	6 (30)	
**Age**				0.395
<65	81	68 (54.84)	13 (65)	
≥65	63	56 (45.16)	7 (35)	
**Undernutrition**				0.955
Without undernutrition	123	106 (85.48)	17 (85)	
With undernutrition	21	18 (14.52)	3 (15)	
**ECOG**				0.993
0	107	92 (74.19)	15 (75)	
1	29	25 (20.16)	4 (20)	
2	8	7 (5.65)	1 (5)	
**Comorbidity**				0.618
0	95	82 (66.13)	13 (65)	
1	39	34 (27.42)	5 (25)	
2	7	5 (4.03)	2 (10)	
3	3	3 (2.42)	0 (0)	
**Location**				0.432
Left-side	121	103 (83.06)	18 (90)	
Right-side	23	21 (16.94)	2 (10)	
**Histological type**				0.297
Adenocarcinoma	75	64 (51.61)	11 (55)	
Mucous adenocarcinoma	2	1 (0.81)	1 (5)	
Unknown	67	59 (47.58)	8 (40)	
**Differentiated grade**				0.226
Moderate	76	68 (54.84)	8 (40)	
Poor	38	33 (26.61)	5 (25)	
Unknown	30	23 (18.55)	7 (35)	
**CRC surgery**				0.877
No	27	23 (18.55)	4 (20)	
Yes	117	101 (81.45)	16 (80)	
**Liver surgery**				0.588
No	116	99 (79.84)	17 (85)	
Yes	28	25 (20.16)	3 (15)	
**Liver metastasis**				0.947
No	71	61 (49.19)	10 (50)	
Yes	73	63 (50.81)	10 (50)	
**Num of metastatic organs**				0.619
<2	79	67 (54.03)	12 (60)	
>=2	65	57 (45.97)	8 (40)	
**RAS**				0.573
Wild	76	67 (54.03)	9 (45)	
Mutation	57	47 (37.9)	10 (50)	
Unknown	11	10 (8.06)	1 (5)	
**BRAF**				0.027
Wild	131	114 (91.94)	17 (85)	
Mutation	3	1 (0.81)	2 (10)	
Unknown	10	9 (7.26)	1 (5)	
**HER2**				0.959
Negative	48	41 (33.06)	7 (35)	
Positive	17	15 (12.1)	2 (10)	
Unknown	79	68 (54.84)	11 (55)	
**MSI**				0.155
MSS	115	98 (79.03)	17 (85)	
MSI-H	6	4 (3.23)	2 (10)	
Unknown	23	22 (17.74)	1 (5)	
**BEV**				0.306
No	80	71 (57.26)	9 (45)	
Yes	64	53 (42.74)	11 (55)	
**CET**				0.416
No	89	75 (60.48)	14 (70)	
Yes	55	49 (39.52)	6 (30)	
**ORR**				0.025
No	63	59 (47.58)	4 (20)	
Yes	56	47 (37.9)	9 (45)	
Unknown	25	18 (14.52)	7 (35)	
**Chemotherapy continuity**				0.716
Continuous	126	108 (87.1)	18 (90)	
Incontinuity	18	16 (12.9)	2 (10)	
**Radiotherapy**				0.394
No	124	108 (87.1)	16 (80)	
Yes	20	16 (12.9)	4 (20)	
**PLT**				0.551
Normal	128	111 (89.52)	17 (85)	
Abnormal	16	13 (10.48)	3 (15)	
**WBC**				0.939
Normal	107	92 (74.19)	15 (75)	
Abnormal	37	32 (25.81)	5 (25)	
**NEU**				0.213
Normal	135	115 (92.74)	20 (100)	
Abnormal	9	9 (7.26)	0(0)	
**Hb**				0.585
normal	93	79 (63.71)	14 (70)	
abnormal	51	45 (36.29)	6 (30)	
**ALT**				0.28
normal	117	99 (79.84)	18 (90)	
abnormal	27	25 (20.16)	2 (10)	
**AST**				0.191
normal	123	104 (83.87)	19 (95)	
abnormal	21	20 (16.13)	1 (5)	
**TBil**				0.841
normal	138	119 (95.97)	19 (95)	
abnormal	6	5 (4.03)	1 (5)	

**Notes:**

Abbreviations: Fu, Fluorouracil; RDI, Relative dose intensity; CRC, Colorectal cancer; MSI, Microsatellite Instability; BEV, Bevacizumab; CET, Cetuximab; ORR, Overall response rate; PLT, Platelet; WBC, White blood cell; NEU, Neutrophil; Hb, Hemoglobin; ALT, Glutamic-pyruvic transaminase; AST, Glutamic-oxalacetic transaminase; TBil, Total bilirubin.

a *P* values obtained from the χ2 test. All statistical tests were two-sided.

### Effect of chemotherapy dose delay on chemotherapy efficacy

**Figure 1:**
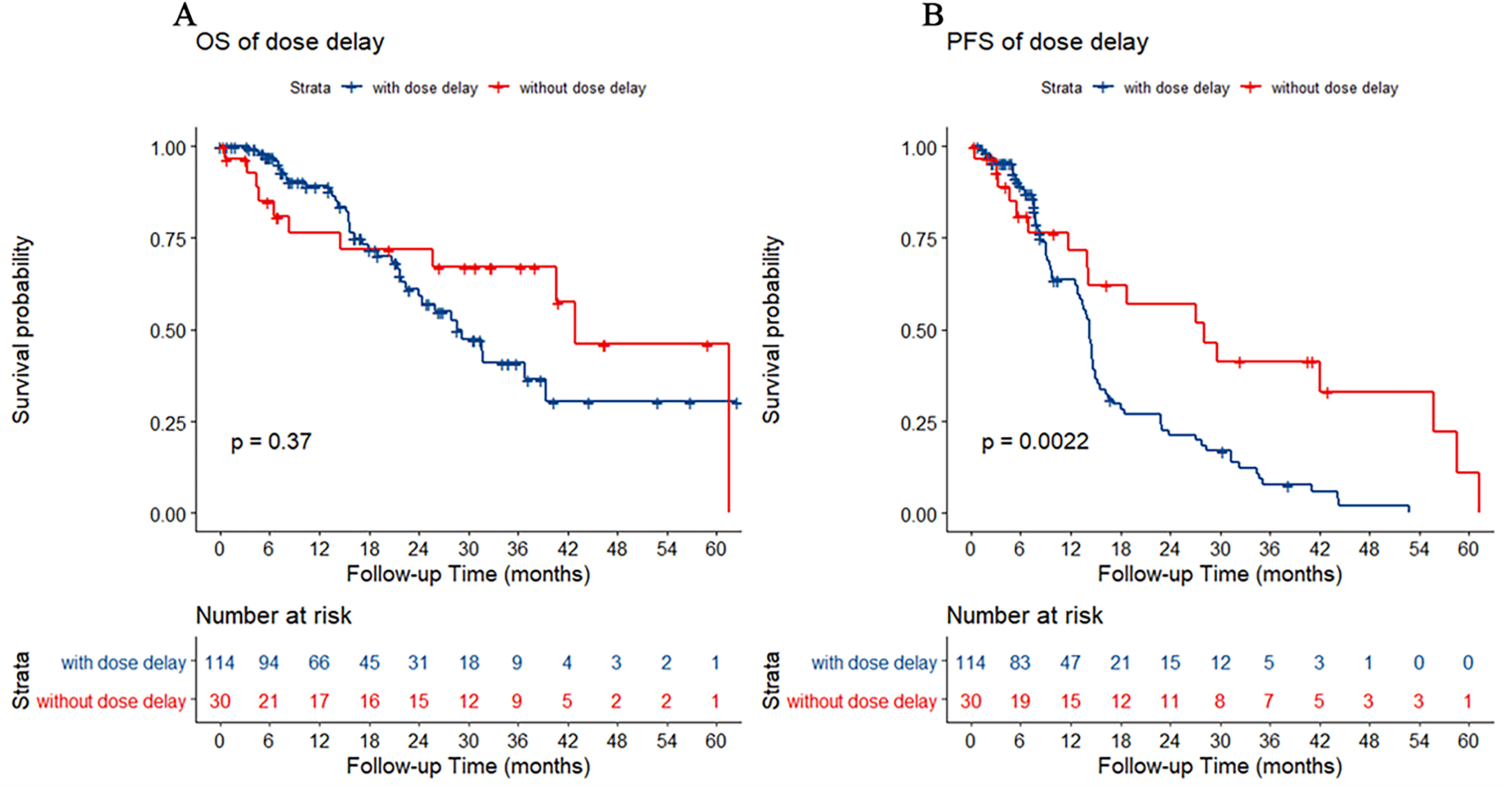
Kaplan–Meier estimate for patients with advanced colorectal cancer (CRC) treated with FOLFIRI based first-line treatment. (A) Overall survival (OS) was measured between advanced CRC patients with or without dose delay (dose delay cutoff value was 3 days, *p* = 0.37). (B) Progression-free survival (PFS) was measured between advanced CRC patients with or without dose delay. Patients without dose delay had better survival (*p* = 0.002).

There were 114 patients with chemotherapy dose delay (dose delay >3 days) and 30 patients without chemotherapy dose delay (dose delay ≤3 days). Between the two groups, there was no statistically significant difference in OS (*p* = 0.37). However, the PFS of patients without a dose delay was better than that of patients with a dose delay (*p* = 0.002) (Fig. 1).

### Effect of 5-Fu dose reduction on chemotherapy efficacy

**Figure 2:**
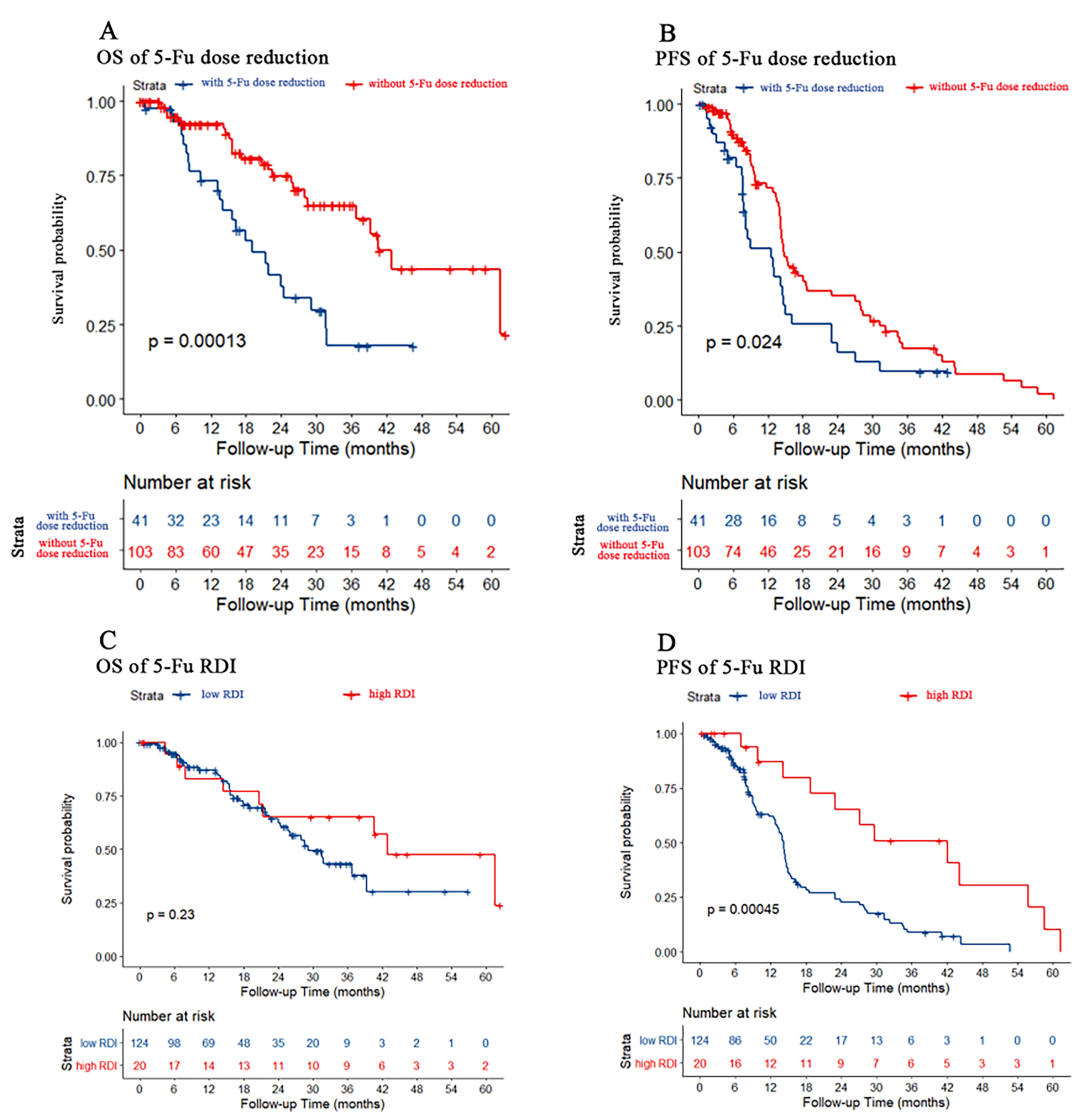
Kaplan–Meier estimate for patients with advanced CRC treated with FOLFIRI based first-line treatment. (A) OS was measured between advanced CRC patients with or without 5-Fu dose reduction [(actual dose/standard dose * 100%) ≤85% was considered dose reduction]. Patients without 5-Fu dose reduction had better OS (*p* < 0.001). (B) PFS was measured between advanced CRC patients with or without 5-Fu dose reduction. Patients without 5-Fu dose reduction had better PFS (*p* = 0.024). (C) OS was measured between advanced CRC patients in the low or high 5-Fu relative dose intensity (RDI, RDI cutoff was 20%, *p* = 0.23). (D) PFS was measured between advanced CRC patients in low or high 5-Fu RDI. Patients in the high RDI group had better PFS (*p* < 0.001).

We used dose reduction and RDI to evaluate the effect of 5-Fu dose reduction on prognosis. As shown in Figs. 2A and 2B, PFS and OS were both better in patients without 5-Fu dose reduction than in patients with 5-Fu dose reduction (*p* < 0.001 and *p* = 0.024, respectively). However, there were no differences in OS for both 5-Fu dose reduction and RDI. As shown in Figs. 2C and 2D, only PFS was better in patients with a high 5-Fu RDI than in patients with a low 5-Fu RDI (*p* < 0.001). However, there was no difference in OS between the high 5-Fu RDI group and the low 5-Fu RDI group (*p* = 0.23).

### Stratified analyses of OS and PFS according to 5-Fu RDI

**Figure 3:**
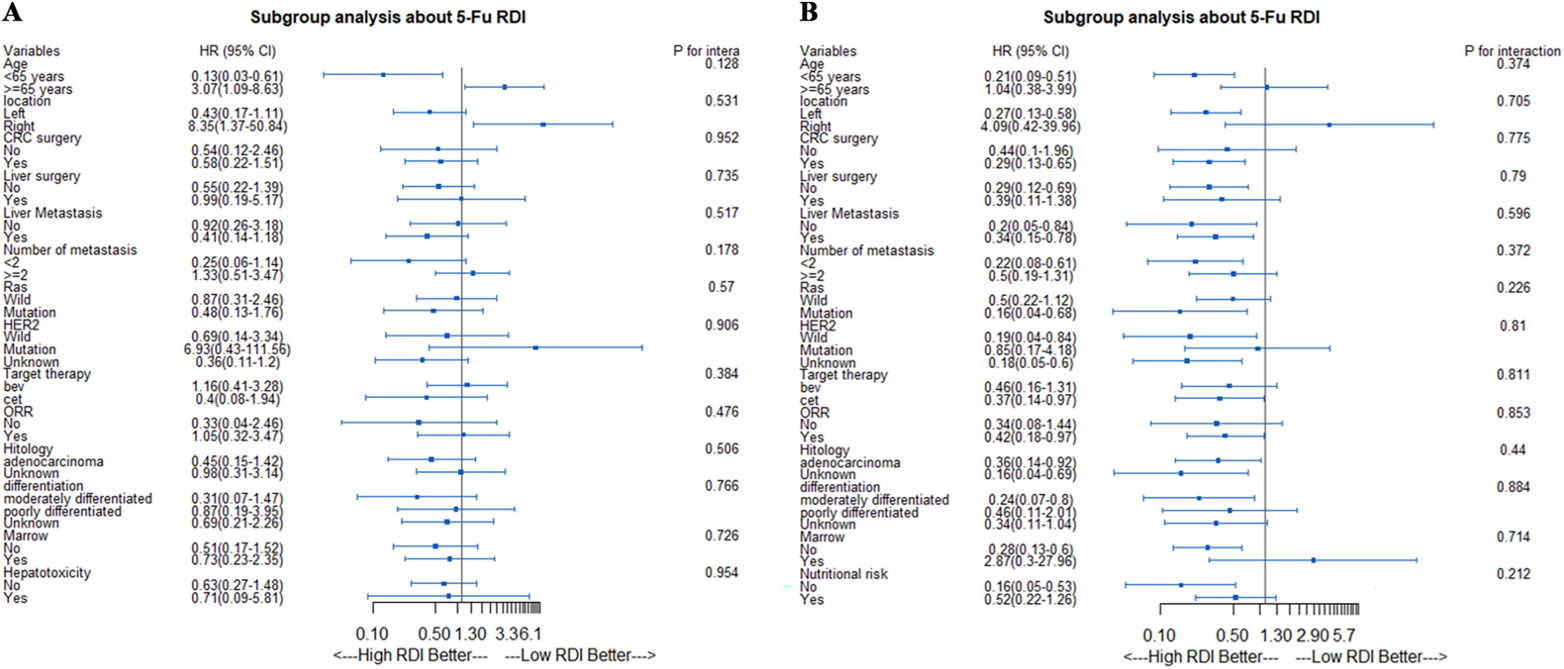
Stratified analyses according to 5-Fu RDI. (A) Stratified analyses of OS according to 5-Fu RDI. (B) Stratified analyses of PFS according to 5-Fu RDI.

In the stratified analysis of OS based on the 5-Fu RDI, we found that in the age <65 years subgroup, patients with a high RDI had better OS. However, in the age ≥65 years subgroup, patients with a low RDI had better OS (Fig. 3A). In the stratified analysis of PFS based on the 5-Fu RDI, patients with a high 5-Fu RDI had better survival in almost all subgroups. However, in the age ≥65 years subgroup, patients with a low RDI tended to have a better PFS (Fig. 3B).

### Effect of 5-Fu dose reduction on chemotherapy efficacy in young and old patients

**Figure 4:**
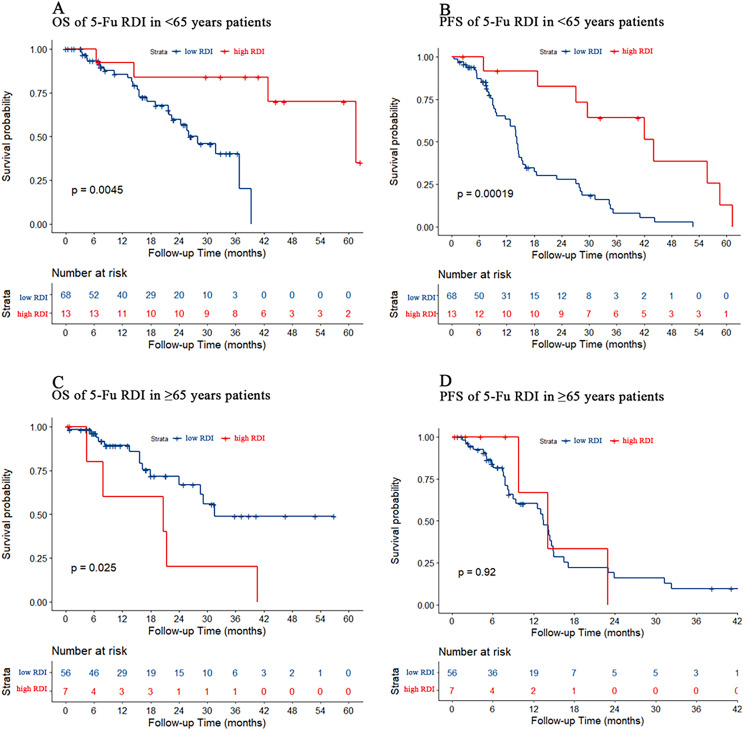
Kaplan–Meier estimate for patients with advanced CRC treated with FOLFIRI based first-line treatment in different age group. (A) OS was measured in <65 years advanced CRC patients between the low and high 5-Fu RDI groups. Patients in the high 5-Fu RDI group had better OS (*p* = 0.005). (B) In <65 years advanced CRC cohort, patients in the high 5-Fu RDI group had better PFS (*p* < 0.001). (C) OS was measured in ≥65 years advanced CRC patients between the low and high 5-Fu RDI groups. Patients in the low 5-Fu RDI group had better OS (*p* = 0.025). (D) In ≥65 years advanced CRC cohort, patients in the high 5-Fu RDI group had similar outcomes to patients in the low 5-Fu RDI group (*p* = 0.92).

Patients younger than 65 years were defined as the young cohort, and those older than 65 years were defined as the older cohort. In the young cohort, PFS and OS were both better in patients with a high 5-Fu RDI than in those with a low 5-Fu RDI (*p* = 0.005 and *p* < 0.001, respectively) (Figs. 4A and 4B). In the older cohort, patients with a low 5-Fu RDI had better OS than those with a high 5-Fu RDI (*p* = 0.025, Fig. 4C). There was no statistically significant difference in PFS for 5-Fu RDI (Fig. 4D).

### Effect of irinotecan dose reduction on chemotherapy efficacy

**Figure 5:**
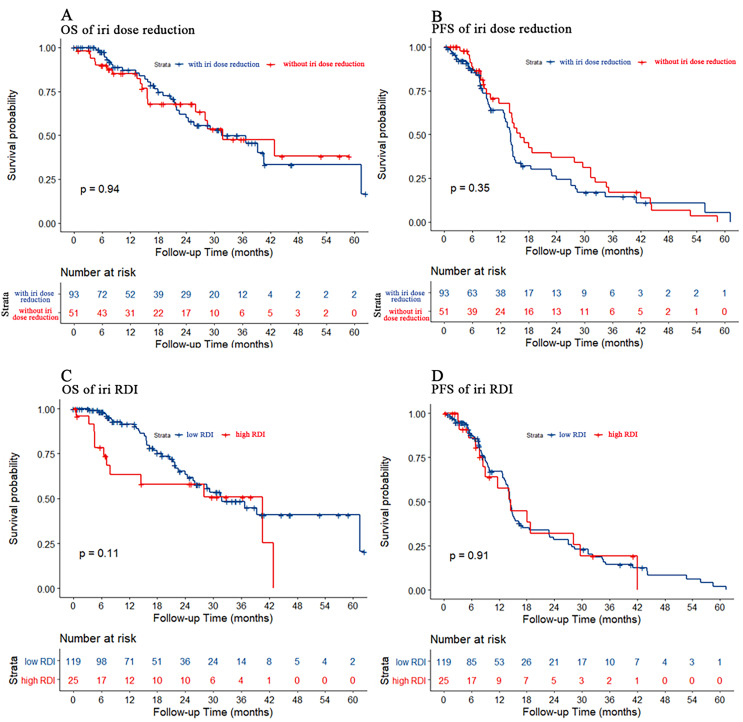
Kaplan–Meier estimate for patients with advanced CRC treated with FOLFIRI based first-line treatment. (A) OS was measured between advanced CRC patients with or without irinotecan dose reduction (*p* = 0.94). (B) PFS was measured between advanced CRC patients with or without irinotecan dose reduction. The prognosis of patients without irinotecan dose reduction were similar to patients with irinotecan dose reduction (*p* = 0.35). (C) OS was measured between advanced CRC patients in the low or high irinotecan RDI group (irinotecan RDI cutoff was 30%, *p* = 0.11). (D) PFS was measured between advanced CRC patients in the low or high irinotecan RDI group. The prognosis of patients in the high irinotecan RDI group were similar to patients in the low irinotecan RDI group (*p* = 0.91).

We also used dose reduction and RDI to evaluate the effect of irinotecan dose reduction on prognosis. As shown in Fig. 5, there were no statistically significant differences in either PFS or OS for either dose reduction or RDI.

## Discussion

The optimal chemotherapy regimen and treatment of mCRC have been the subject of many retrospective and prospective studies in the past decade. Due to personal or disease reasons and treatment-related effects, it is common to modify the chemotherapy dose and interval of the chemotherapy cycle. Incidences of dose delays, dose reduction, missing doses, and reduced RDI were higher in those with CRC than in those with breast cancer, NHL and non-small cell lung cancer (NSCLC) (Denduluri et al., 2015). Reduced RDI was common among old patients with CRC who received adjuvant or neoadjuvant chemotherapy. Older patients may have poor tolerance and compliance to chemotherapy (Denduluri et al., 2015). However, few studies have focused on the impact of chemotherapy dose modification in metastatic CRC patients. To our knowledge, this is the first study to analyse the correlation between first-line chemotherapy dose or schedule modification and survival in mCRC patients.

In clinical practice, dose reduction and delay of cytotoxic agents usually lead to a reduction in RDI, resulting in adverse events. In our research, a delay of more than 3 days was associated with significantly poorer PFS, leading to a significantly increased risk of disease progression. However, there was no statistically significant difference in OS. Haematological toxicity was the most common reason for dose delays (Lyman, Abella & Pettengell, 2014). Our findings are consistent with the findings of similar studies conducted in other cancer sites (Joseph et al., 2015; Starbuck et al., 2018). In many previous studies, dose delay was defined as chemotherapy administration delay ≥3 or ≥7 days from the intended date (Crawford et al., 2020; Denduluri et al., 2015). It is likely that if more prolonged cut-offs were utilized, the adverse effect of dose delay would have been greater. Our current data demonstrated the importance of avoiding dose delay if at all possible.

In this study, we evaluated the relationships between the RDI of irinotecan and 5-Fu and survival outcomes, specifically the impact of dose reduction on PFS and OS in patients receiving FOLFIRI. We found that the dose reduction and RDI of 5-Fu might have a stronger association with survival than that of irinotecan. Patients with mCRC who received an RDI > 80% of irinotecan had no significant difference from those who received an RDI ≤ 80% in either PFS or OS. However, we found that a 5-Fu dose reduction >85% was a significant predictor of PFS and OS. Furthermore, a higher RDI of 5-Fu had a significant positive impact on PFS. Therefore, we conclude that irinotecan dose reduction may have less impact on survival than 5-Fu for patients receiving FOLFIRI-based first-line chemotherapy. If drug dose modification is carried out, the dose of irinotecan may be reduced first, rather than 5-Fu. However, it is important to be cautious in dose reduction because the number of patients enrolled in our study was too small. Additionally, doctors and patients should try to avoid extending the chemotherapy interval. When the chemotherapy is delayed, it will become a 3-week regimen. This issue should be further explored in a larger dataset.

In contrast to our results, a study from Japan found that a higher RDI of irinotecan had a significant positive impact on the objective response rate (ORR), disease control rate (DCR), PFS and OS. Dose reduction of irinotecan was an independent prognostic factor for PFS, while dose delay was not significantly associated with PFS (Nakayama et al., 2014). However, we found that reducing the RDI of irinotecan did not affect the curative effect on tumour response and survival outcomes. Previous evidence suggests that polymorphisms homozygous for UGT1A1 might be associated with increased incidences of serious haematological or gastrointestinal toxicities (Satoh et al., 2011). Several dose escalation studies found that the dose of irinotecan should be guided by the UGT1A1 genotype for patients with gene mutations to reduce the dose without affecting the effect of irinotecan (Xu et al., 2018). Dose modification of irinotecan based on UGT1A1 polymorphisms might explain these results. However, most studies showing adverse consequences of dose modification were basically retrospective, and the sample size was limited.

Some studies have confirmed that age-related comorbidities (Delgado-Ramos et al., 2020), physiological changes, female sex, obesity with a body surface area (BSA) >2.0 m^2^ and the progressive loss of systemic protein or muscle (Cespedes Feliciano et al., 2017) will increase the risk of chemotherapy-induced toxicity. Many studies have found that older age was a risk factor for the development of chemotherapy-associated toxicities (Neugut et al., 2006; Søgaard et al., 2013; Shayne et al., 2007, 2009). Chemotherapy-induced neutropenia usually leads to hospitalization and the need for intravenous antibiotics. Neutropenia may result in dose reduction, delay, or even discontinuation of chemotherapy, which in turn may affect the prognosis of patients (Lyman, Abella & Pettengell, 2014). Our research revealed that 5-FU dose adjustment has a significant impact on prognosis and survival. Moreover, PFS was significantly different between low and high RDI of 5-FU. However, there were no differences in OS. From subgroup analysis, patients in the low 5-Fu RDI group were associated with poor PFS and OS in the young age (<65 years) population. However, this difference was not observed in the older group (≥65 years). Increased age and comorbidities, as well as the toxicity of 5-FU, may also lead to a reduction in chemotherapy completion, and a shorter duration of chemotherapy is related to poorer survival in mCRC. This tells us that chemotherapy dose delay and reduction need to be performed with caution in young patients.

Studies that evaluated the impact of dose reduction and delays on patient outcomes have been inconsistent to date. Research by Starbuck et al. (2018) and Olawaiye et al. (2018) showed that on-time completion of chemotherapy is associated with improved survival and higher complete response rates in epithelial ovarian cancer (EOC). A delayed completion of chemotherapy was associated with decreased survival. This view is similar to our research. However, the study did not consider the effect of dose modification on prognosis. However, some studies investigated the effect of dose reduction and/or delay and found no significant difference in survival for any group in EOC (Nagel et al., 2012; Sivakumaran et al., 2020). Due to the limited sample size of this study, we cannot provide more convincing information. In addition, a retrospective study explored whether chemotherapy delay alone was associated with decreased survival in the HER2-positive breast cancer subtype (Liutkauskiene et al., 2018). This finding establishes the importance of molecular typing and suggests that it may have an impact on the results. We expect larger sample data to explore the correlation between chemotherapy delay and RAS gene status. Additionally, we may have overlooked the impact of targeted drugs on prognosis and survival. Although the patients who have been successfully treated by resection of metastases or who have benefited from local treatment and radiotherapy have experienced chemotherapy delay, the above treatments have improved the prognosis of the patients. The benefits of these treatments may interfere with the correlation between RDI and prognosis. Further work and large samples are needed to elucidate the correlation between RDI and survival.

In conclusion, our study confirms the importance of maintaining the schedule of chemotherapy regimens. In the real world, it is important to reduce the occurrence of chemotherapy delay as much as possible. We found that the RDI of 5-Fu might have a stronger association with survival than that of irinotecan. Especially among patients younger than 65 years, 5-Fu doses need to be modified with caution. Further work is needed to clarify the role of dose delay and reduction in chemotherapy administration.

## Supplemental Information

10.7717/peerj.15995/supp-1 Supplemental Information 1 Raw data.

## Additional Information and Declarations

### Data Availability

The following information was supplied regarding data availability: The raw measurements are available in the Supplemental File.
